# HDAC2 as a target for developing anti-cancer drugs

**DOI:** 10.1016/j.csbj.2023.03.016

**Published:** 2023-03-13

**Authors:** Hyein Jo, Kyeonghee Shim, Han-Ul Kim, Hyun Suk Jung, Dooil Jeoung

**Affiliations:** Department of Biochemistry, College of Natural Sciences, Kangwon National University, Chuncheon 24341, Republic of Korea

**Keywords:** CAGE, cancer associated gene, DNMT1, DNA methyl transferase 1, EMT, epithelial to mesenchymal transition, GC, gastric cancer, HCC, hepatic cell carcinoma, HDAC2, histone deacetylase 2, miRNAs, micro RNAs, NSCLC, non-small cell lung cancers, PTEN, phosphatase and tensin homolog deleted on chromosome 10, Anti-cancer drugs, Anti-cancer drug resistance, Histone deacetylase 2, Prognostic marker, Tumorigenesis

## Abstract

Histone deacetylases (HDACs) deacetylate histones H3 and H4. An imbalance between histone acetylation and deacetylation can lead to various diseases. HDAC2 is present in the nucleus. It plays a critical role in modifying chromatin structures and regulates the expression of various genes by functioning as a transcriptional regulator. The roles of HDAC2 in tumorigenesis and anti-cancer drug resistance are discussed in this review. Several reports suggested that HDAC2 is a prognostic marker of various cancers. The roles of microRNAs (miRNAs) that directly regulate the expression of HDAC2 in tumorigenesis are also discussed in this review. This review also presents HDAC2 as a valuable target for developing anti-cancer drugs.

## Graphical Abstract

**Figure:**
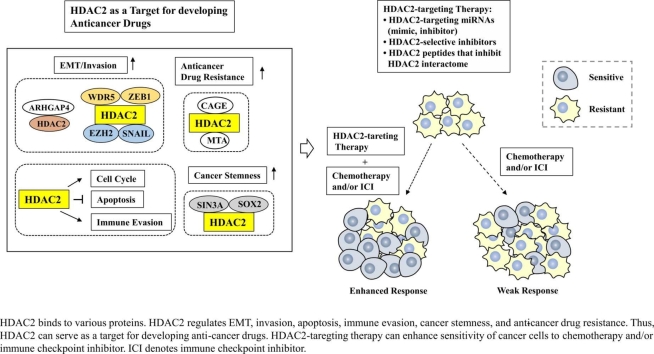
HDAC2 binds to various proteins. HDAC2 regulates EMT, invasion, apoptosis, immune evasion, cancer stemness, and anti-cancer drug resistance. Thus, HDAC2 can serve as a target for developing anti-cancer drugs. HDAC2-targeting therapy can enhance sensitivity of cancer cells to chemotherapy and/or immune checkpoint inhibitor. ICI denotes immune checkpoint inhibitor.

## 1 Introduction

HDACs can remove the acetyl group from the lysine residue at the N-terminal region of histone cores (H2A, H2B, H3, and H4), allowing DNA to wrap histone more tightly [1]. HDAC proteins are classified into four groups based on function and DNA sequences. Class I, II, and IV are inhibited by trichostatin A (TSA) and have a zinc-dependent active site. Class III HDACs are a family of nicotine amide dinucleotide-dependent proteins known as sirtuins and are not affected by TSA. Class II HDACs (HDAC4, 5, 6, 7, 9, and 10) can shuttle in and out of the nucleus. Unlike other HDACs, HDAC6 is a cytoplasmic protein and deacetylates tubulin, hsp90, and cortactin. Class I HDACs, such as HDAC1, HDAC2, HDAC3, and HDAC8, have been well studied. They are known to interact with various transcription factors (TFs) [2], [3]. HDAC 1, 2, and 3 are found primarily in the nucleus, whereas HDAC8 is found in both the nucleus and the cytoplasm.

**Fig. 1:**
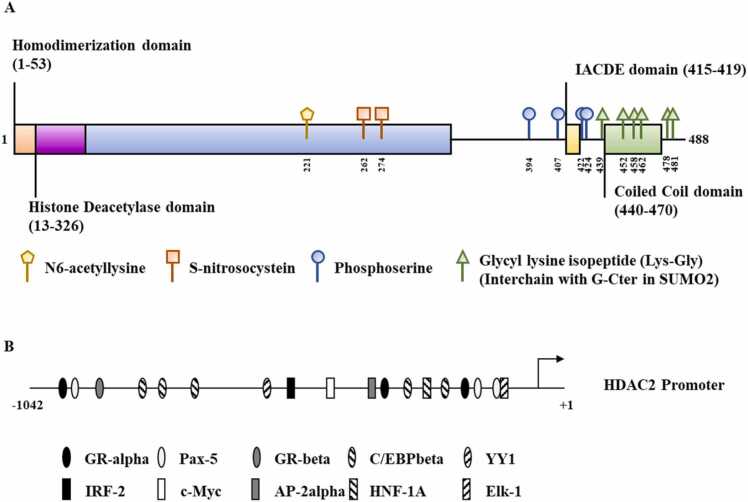
Functional domains and expression regulation of HDAC2. (A) Functional domains of HDAC2. Targets of posttranslational modifications are shown. SUMO, small ubiquitin-like modifier. (B) Potential binding sites for TFs in the promoter sequences of HDAC2. +1 denotes transcription start site. → denotes direction of transcription. C/EBP, CCAAT box enhancer binding protein; Elk-1, ets-like protein 1; GR, glucocorticoid receptor; HNF, hepatocyte nuclear factor; IRF2, Interferon regulatory factor 2.; YY1, yin yang 1.

HDAC2, a member of the class I HDACs, has been reported to play important roles in cellular proliferation, cell signaling, cancer initiation and progression, inflammation, and gene expression regulation. HDAC2 mostly targets histones H3 and H4. HDAC2 is comprised of a conserved homodimerization domain, an HDAC domain, an IACDE domain (amino acids 415–419), and a coiled-coil domain (Fig. 1A). Although HDAC2 is known as a protein modifier, HDAC2 is also widely modified. These modifications of HDAC2 include acetylations [4], phosphorylations [5], nitosylations [6], and sumoylations [7]. Fig. 1A shows the amino acid residues of HDAC2 that undergo posttranslational modifications. Fig. 1B shows potential binding sites for TFs in the promoter sequences of HDAC2. C-MYC was shown to directly increase the expression of HDAC2 by binding to the promoter sequences of HDAC2 [8]. Yin yang 1 (YY1) bound to HDAC2 and regulated the sensitivity of clear cell renal cell carcinoma (ccRCC) to tyrosine kinase inhibitors in ccRCC [9]. Given that HDAC2 can regulate the expression of various genes, HDAC2 might contribute to the pathogenesis of various diseases. This review summarizes the roles of HDAC2 in tumorigenesis and anti-cancer drug resistance. It also presents HDAC2 as a prognostic marker of various cancers. The roles of miRNAs that can regulate the expression of HDAC2 are presented and HDAC2-regulated miRNAs are also presented. Overall, HDAC2 can be a valuable target for developing anti-cancer drugs.

## 2 The role of HDAC2 in tumorigenesis

HDAC2 was overexpressed in lung cancer tissues compared to adjacent normal tissue [10]. Expression levels of HDACs, including HDAC2, were upregulated in metastatic human colorectal cancer (CRC) [11]. The expression of HDAC2 was higher in non-functioning pituitary adenomas (NFPAs) than in normal pituitary tissue [12]. Class I HDAC knockout of individual HDACs, such as HDAC1 and HDAC2, inhibited invasiveness and blocked local tumor growth in xenografted mice [13]. Phosphoribosylaminoimidazole carboxylase and phosphoribosylaminoimidazole succinocarboxamide synthetase (PAICS) was upregulated in gastric cancer (GC). High levels of PAICS were correlated with the poor prognosis of patients with GC [14]. The downregulation of PAICS suppressed GC growth both in vitro and in vivo [14]. PAICS interacted with histone deacetylases HDAC1 and HDAC2 and enhanced DNA damage repair by increasing the expression of RecA-like recombinase RAD51 [14]. The expression level of ubiquitin-specific protease 19 (USP19) was decreased in several types of tumors [15]. USP19 could modulate DNA damage repair by deubiquitinating HDAC2, which resulted in genomic instability and contributed to tumorigenesis [15]. The downregulation of HDAC2 inhibited peroxisome proliferator activated receptor γ (PPARγ) signaling, glycolysis, and lipogenesis to suppress the growth of HCC [16]. PPARγ is known to play an important role in the control of glucose homeostasis [16]. The decreased expression of fructose bis-phosphatase 1 (FBP1), a rate-limiting enzyme in gluconeogenesis, was associated with the poor prognosis of patients with HCC [17]. The low expression of FBP1 was correlated with high levels of HDAC2 in the tissues of patients with HCC [17]. This implies that high levels of HDAC2 could predict the poor prognosis of patients with HCC and suggests the role of HDAC2 in cancer initiation and progression. It is also probable that HDAC2 could be a prognostic cancer marker.

## 3 High level of HDAC2 can predict poor prognosis of various cancers

The expression level of HDAC2 was increased in osteosarcoma (OS) cells and tissues. High levels of HDAC2 could predict the poor prognosis of OS patients [18]. High levels of HDAC2 are associated with the poor survival of patients with low-grade and early-stage HCC [19]. High levels of HDAC2 could predict the poor prognosis of patients with HCC [20]. Spalt-like transcriptional factor 4 (SALL4), a stem cell marker, was reactivated in several cancers and interacted with HDAC2 [21]. High HDAC2 expression (46%, 62/135) was associated with poor histologic differentiation and a lower 5-year survival rate of patients with HCC [21]. The co-expression of SALL4 with HDAC1 and/or HDAC2 was correlated with low expression levels of PTEN [21]. High levels of HDAC2 expression could predict the poor prognosis of patients with CRC [22].

HDAC2 levels were significantly higher in oral squamous cell carcinoma (OSCC) and pre-oral cancer patient groups than in controls [23]. High levels of HDAC2 were associated with tumor-node-metastasis stages of OSCC patients [23]. The expression level of HDAC2 was higher in esophageal squamous cell carcinoma (ESCC) tissues than in atypical hyperplasia tissues [24]. The expression of HDAC2 was closely associated with histological grade, and lymph node metastasis [24].

High levels of HDAC2 were closely associated with the overexpression of human epidermal growth factor receptor 2 and nodal metastasis in breast cancer [25]. High levels of HDAC2 were reported in aggressive basal-like breast cancer and could predict a poor prognosis in breast cancer patients [26]. Mocetinostat, an HDAC inhibitor, showed anti-tumor effects in HDAC2-overexpressing basal-like breast cancer lines [26]. In breast cancer, high levels of HDAC2 were closely correlated with lymph node metastasis and high levels of multidrug resistance protein [27]. High levels of HDAC2 could predict the poor prognosis of breast cancer patients who received chemotherapy containing anthracyclines [27].

High levels of HDAC2 were correlated with a high incidence of lymph node metastasis in human gallbladder carcinoma (PGC) [28] and could predict a poor prognosis in patients with cholangiocarcinoma (CCA) [29]. Two HDAC inhibitors, TSA and vorinostat, suppressed proliferation of CCA cells by decreasing the expression of transforming acidic coiled-coil-containing protein 3 (TACC3) [29]. High levels of TACC3 could predict a poor prognosis in CCA patients [29]. Thus, TACC3 might be a useful prognostic biomarker for CCA.

Hdac1/2-deficient epidermis displayed elevated levels of acetylated p53 and the increased expression of the senescence gene p16 [30]. Hdac1/2 deletion reduced proliferation and increased apoptosis [30]. The knockout of either p53 or p16 partially rescued both proliferation and basal cell viability [30]. Table 1 shows the close relationships between HDAC2 expression and the clinicopathological features of cancer patients. This shows the potential of HDAC2 as a cancer prognosis marker.

**Table 1:** Association of HDAC2 expression with the clinicopathological features of cancer patients.

Tumor types	Clinical parameter (s)	HDAC2 expression		Clinical significance	Ref.
		Low (n)	High (n)		
Hepatocellular carcinoma	Overall survival (OS) Disease-free survival (DFS)	54	46	P < 0.05 (OS) P < 0.05 (DFS)	[20]
Colorectal cancer	Liver metastasis No Yes	51 Yes; 4/51 No;47/51	70 Yes; 24/70 No; 46/70	P = 0.001	[22]
Esophageal squamous cell carcinoma	Lymph node metastasis	Yes; 1/31 No;13/38	Yes; 30/31 No;25/38	P = 0.001	[24]
Breast cancer	Histological grades; G1/G2/G3	G1;34/60 G2;39/92 G3;17/55	G1;6/60 G2; 21/92 G3;24/55	P < 0.001	[25]
Breast cancer	Overall survival	600	360	P = 0.0018	[26]
Breast cancer	Lymph node metastasis	Yes; 41/121 No;53/94	Yes;80/121 No; 42/94	P = 0.002	[27]
Gallbladder cancer	Clinical stages	I; 28/46 II; 10/52 III; 4/18 IV; 2/20	I; 18/46 II; 42/52 III; 14/18 IV;17/20	P = 0.005	[28]
Cholangiocarcinoma	TNM stages	I-II;29/45 III-IV; 8/34	I-II;16/45 III-IV; 26/34	P < 0.001	[29]

## 4 The mechanism of HDAC2-promoted tumorigenesis

### 4.1 Effects of HDAC2 on signaling

HDAC2 regulated the expression of receptor tyrosine kinases, including platelet growth factor receptor α (PDGFRα), PDGFRβ, and epidermal growth factor receptor (EGFR) [31]. Epidermal growth factor (EGF) increased the expression of creatine kinase α (CK2α), which in turn, increased the expression of HDAC2 in HCC [32]. Thus, HDAC2 might mediate EGFR signaling to promote cancer cell proliferation. HDAC2 suppressed the anti-oncogenic transforming growth factor β signaling pathway and mediated pancreatic ductal adenocarcinoma metastasis [31]. The SIN3 transcriptional regulator family 3 A (SIN3A)-HDAC1/2 transcription repressor complex silenced bone morphogenetic protein 6 (BMP6) expression, which increased the metastatic potential and oncogenic potential of melanoma cells by suppressing BMP6-activated mothers against DPP homolog 5 signaling [33]. G-protein-coupled receptor 126 (GPR126) was overexpressed in CRC tissue compared to normal adjacent colon tissues [34]. The downregulation of GPR126 inhibited the proliferation and tumorigenic potential of CC cells by decreasing the expression of HDAC2 [34].

### 4.2 Effects of HDAC2 on EMT, invasion, and migration

Endothelial-mesenchymal transition (EndMT) is initiated by the recruitment of aberrantly phosphorylated DNMT1 to the RAS protein activator-like 1 CpG island promoter by HDAC2, causing aberrant promoter methylation and transcriptional suppression, leading to increased Ras-GTP activity and the activation of the common EndMT regulators TWIST and zinc finger protein SNAIL [35]. Enhancer of zeste homolog 2 (EZH2) was upregulated in several types of human cancers. The EZH2/HDAC2/SNAIL complex bound to E-cadherin (CDH1) promoter sequences to repress the expression of CDH1 [36]. High levels of WD repeat domain 5 (WDR5) could predict the poor prognosis of patients with CCA [37]. WDR5 increased the levels of hypoxia-inducible factor 1 subunit α (HIF-1α) by binding to HDAC2, which enhanced EMT [37].

Zinc finger E-box-binding homeobox 1, a TF, recruited HDAC complexes to CDH1 promoter sequences to decrease the expression of CDH1 in pancreatic cancer (PC) cells [38]. HDAC2 enhanced the migration and invasion of NSCLC cells by upregulating fibronectin in a nuclear factor- κB (NF-κB)-dependent manner [39]. HDAC2 enhanced HIF-1α stability, which, in turn, enhanced the cell invasion and migration of oral squamous cancer cells [40]. Rho GTPase-activating protein 4, a new regulator of the β-catenin pathway, interacted with and ubiquitinated HDAC2, which, in turn, inhibited β-catenin activation to decrease the invasion and migration of PC cells [41].

### 4.3 Effects of HDAC2 on apoptosis and cell cycle progression

Decreased nuclear receptor-binding protein 2 (NRBP2) expression was observed in thyroid cancer (TC) tissues and cells [42]. Low levels of NRBP2 predicted the poor prognosis of patients with TCs [42]. GATA family of TF1 GATA-binding protein 1 (GATA1) recruited HDAC2 to NRBP2 promoter sequences to suppress the expression of NRBP2 [27]. NRBP2 overexpression suppressed the growth of TC cells, M2 polarization of macrophages, and angiogenesis in TC [42].

HDAC1 and HDAC2 contributed to maintaining the expression of p53 mutants in human and genetically defined murine PC cells [43]. The downregulation of HDAC2 inhibited cellular proliferation in a p53-dependent manner in breast cancer cells [44]. HDAC2 inactivation suppressed lung cancer cell growth by increasing the hypophosphorylation of pRb (retinoblastoma) protein and activating p53 and Bax while suppressing Bcl2 [45]. HDAC2 also directly decreased p21WAF1/cyclin-dependent kinase inhibitor 1(CIP1) expression in a p53-independent manner [45]. ΔNp63α, a member of the p53 family of TFs, associated with HDAC1 and HDAC2, formed an active transcriptional repressor complex to mediate the cellular survival of squamous cell carcinoma by repressing the expression of p53-upregulated modulator of apoptosis [46].

Depletion of HDAC2 in PC cell lines enhanced the sensitivity of PC cells to tumor necrosis factor-related apoptosis-inducing ligand (TRAIL) by increasing the expression of TRAIL receptor 1 (DR5), caspase 8 activity, and cleavage of BH3-only protein Bid [47]. HDAC2 promoted the proliferation of HCC by decreasing the expression of PTEN [48]. HDAC2 directly repressed the expression of p21 and p57 to promote G1-to-S-phase cell cycle transition [49]. T-box TF TBX3 recruited HDAC2 to decrease the expression of p57^KIP2^, which, in turn, promoted the proliferation of papillary thyroid carcinoma cells [50]. High levels of FKBP3, a member of FK506-binding proteins (FKBPs), could predict the poor prognosis of patients with NSCLCs [51]. The downregulation of FKBP3 decreased the expression of HDAC2 but increased the expression of p27, which, in turn, inhibited the proliferation of NSCLC [51]. HDAC2 promoted hepatocellular carcinoma cell proliferation by increasing the expression of cell cycle-related proteins, such as cyclin D1, cyclin-dependent kinase 4 (CDK4), and phospho-Rb [52].

### 4.4 Effects of HDAC2 on cancer stemness and immune evasion

**Fig. 2:**
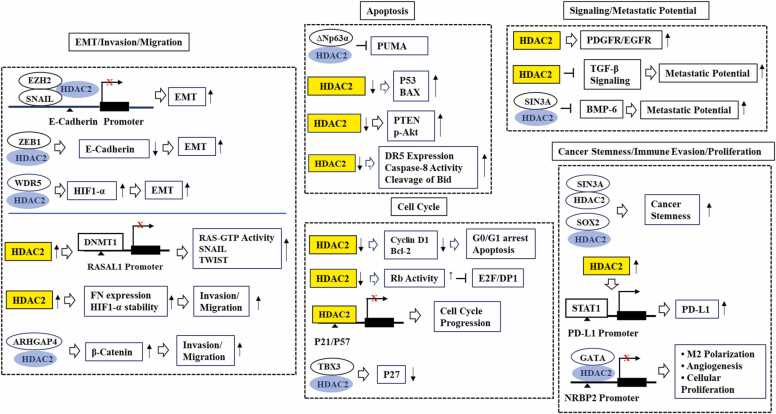
Mechanism of HDAC2-promoted cancer cell proliferation. HDAC2 promotes cancer cell proliferation by regulating various life processes such as EMT, invasion/migration, apoptosis, cell cycle, signaling, cancer stemness, immune evasion, and cellular proliferation. The hollow arrows denote direction of reaction. T bar arrows denotes negative regulation. ↓denotes decreased expression or activity. ↑denotes increased expression or activity. X denotes suppression of transcription.

SIN3A/HDAC corepressor complex, enriched in the homeobox TF NANOG interactome, maintained embryonal stem cell pluripotency and promoted the generation of induced pluripotent stem cells [53]. This implies that HDAC2 could contribute to the stem cell-like properties of cancer cells [53]. Sex-determining region Y-box-containing TF2, a marker of cancer stemness, was associated with HDAC2 [54]. This implies the role of HDAC2 in cancer stemness. HDAC2 and programmed death ligand-1 (PD-L1) expression levels were significantly higher in triple negative breast cancer (TNBC) than in non-TNBC [55]. HDAC2 increased the expression of PD-L1 by increasing the phosphorylation of Janus kinase 1(JAK1), JAK2, and signal transducer and activator of T cells (STAT1), and the recruitment of STAT1 to PD-L1 promoter sequences [55]. The genetic deletion of HDAC2 in TNBC cells inhibited cell proliferation, migration, and cell cycle progression [55], suggesting that HDAC2 could enhance tumorigenesis by promoting immune evasion through PD-L1. Thus, HDAC2 could promote cancer cell proliferation and tumorigenesis by regulating the cell cycle, tumor suppressor genes, EMT, and cancer stem cell-like properties. Fig. 2 shows the mechanisms of HDAC2-promoted cancer cell proliferation.

## 5 Effect of HDAC2 downregulation and inhibition on tumorigenesis

The downregulation of HDAC2 induced the expression of E3 ubiquitin-protein ligase c-Cbl in lung cancer cells [56]. The ectopic expression of c-Cbl decreased EGFR expression and inhibited the growth of NSCLC [56]. HDAC2 expression was upregulated in different histopathologic grades of human GC tissue and GC cell lines [57]. The downregulation of HDAC2 induced G1-S cell cycle arrest and autophagy and restored the activity of the cell cycle regulator p16 [57].

Not all HDAC isoforms are abnormally expressed in certain cancers [58]. Therefore, isoform-selective HDAC inhibition can be one of the best cancer therapeutic strategies. The aberrant expression of HDAC2 contributed to the cancer progression by decreasing the expression of apoptotic proteins, such as phorbol-12-myristate-13-acetate-induced protein 1 and apoptotic peptidase-activating factor 1 [58]. Thus, HDAC2-specific inhibitor could be developed as anti-cancer drugs. As specific HDAC1/2 inhibitors, romidepsin and givinostat suppressed the proliferation of urothelial carcinoma cells by inducing non-apoptotic cell death [59]. Treatment of dedifferentiated liposarcoma cell lines with romidepsin reduced tumor growth and decreased mouse double minute 2 (MDM2) expression. The inhibition of HDAC2 decreased the expression of MDM2 and induced apoptosis in liposarcoma [60]. HDAC2, a downstream effector of Hedgehog (Hh) signaling, was upregulated in medulloblastoma [61]. The inhibition of HDAC2 by mocetinostat induced the apoptosis of medulloblastoma cells by inhibiting Hh signaling [61]. The selective inhibition of HDAC2 by vorinostat decreased the expression of survivin by activating p53 mediated by the downregulation of MDM2 in lung cancer cells [62].

HDAC2 expression was increased in endometrial stromal sarcoma (ESS) compared to adjacent non-neoplastic endometrial stroma [63]. Valproic acid (VPA), an HDAC2-specific inhibitor, decreased the expression of HDAC2 and inhibited G1-S cell cycle transition by increasing the expression of the cell cycle regulator p21 [63]. High levels of HDAC2 were positively associated with the poor prognosis of patients with GC [64]. VPA induced apoptosis by inhibiting Akt signaling and inducing autophagy in GCs [64]. VPA increased acetyl-histone H3 and p21WAF1 levels by upregulating p27, caspase 3, and caspase 9, and downregulating cyclin D1 and survivin in GC cells [65]. The binding of VPA to HDAC2 enhanced the sensitivity of melanoma cells to radiation [66]. VPA suppressed breast cancer cell migration by decreasing the expression of survivin [67]. CUDC-907, a dual inhibitor of HDACs and the phosphatidyl inositol-3 kinase (PI3K)/AKT pathway, suppressed tumor growth by decreasing the expression levels of HDAC2, p-AKT, and p-ERK1/2 [68]. Protocatechualdehyde induced apoptosis in human CC cells by decreasing the expression levels of HDAC2 and cyclin D1 [69]. HDAC2 expression was increased in ESS [70]. HDAC2 inhibition induced G1-S transition arrest by regulating the expression levels of p21 and cyclin D1 [70].

Wogonin exerted anti-cancer effects by inducing the degradation of c-MYC, s-phase kinase -associated protein 2, HDAC1, and HDAC2 [71]. C-MYC increased the expression level of HDAC2 in PC cells [72], implying that targeting HDAC2 might overcome c-MYC-induced oncogenic effects. Trichodermin, an inhibitor of eukaryotic protein synthesis, induced apoptosis by decreasing HDAC2 expression and levels of phosphorylated STAT3 and NF-κB [73].

The effect of 1,25(OH)2D3 on PTEN expression was reversed by overexpressing HDAC2 [74]. 1,25(OH)2D3 inhibited HCC growth by upregulating HDAC2-mediated PTEN and inhibiting the PI3K/Akt signaling pathway [74]. In synovial sarcoma, HDAC2 inactivation induced the degradation of the SS18-SSX driver oncoprotein to inhibit cancer cell proliferation [75]. Thus, the inhibition and downregulation of HDAC2 suppressed cancer cell proliferation by regulating the cell cycle, apoptosis, signaling, and metabolic pathways.

## 6 The molecular network of HDAC2 and miRNAs

miRNAs are small noncoding RNAs that can regulate the expression of genes involved in carcinogenesis [76], [77]. miRNAs can function as oncogenes and tumor suppressors. miRNAs bind to the 3‘-untranslated region (UTR) of target genes to regulate the expression of these target genes. Low levels of miR-31 could predict the poor prognosis of HCC patients [78]. miR-31 directly decreased the expression of HDAC2 in HCC cells [78]. miR-31 expression was significantly decreased in GC tissues and cell lines [78]. Overexpression of miR-31 suppressed proliferation and induced early apoptosis in GC cells by targeting HDAC2 [79]. miR-122–5p inhibited the proliferation of hepatic stellate cells (HSC) by suppressing the proto-oncogene c-Abl/HDAC2 pathway [80]. miRNA profiling analysis revealed that miR-145 targeted HDAC2 and inhibited the growth of HCC [81]. Thus, an miR-145 mimic could be developed as an anti-cancer drug targeting HDAC2-expressing cancers. miR-155 decreased the expression of erythroblastic oncogene B by targeting HDAC2 in breast cancer cells [82]. This implies the role of HDAC2 as a downstream effector of EGFR signaling. The expression of an oncogenic long non-coding RNA (CRNDE) was upregulated in NSCLC tissue, whereas miR-455–3p was downregulated [83]. The downregulation of CRNDE inhibited lung cancer progression and decreased the migration and invasion of NSCLC cells by decreasing the expression of miR-455–3p, which targeted HDAC2 [83]. miR-455–3p expression was downregulated in HCC tissues compared to adjacent normal tissues, and miR-455–3p inhibited cell proliferation but promoted apoptosis in HCC cell lines by decreasing the expression of HDAC2 [84]. miR-455–3p targeted HDAC2 to inhibit cell proliferation and migration and promote cell apoptosis by activating the p53 pathway in prostate cancer (PC) [85]. miR-500a-5p displayed a tumor suppressor role in CRC by targeting the p300/ YY1/HDAC2 axis [86]. HDAC2 was significantly upregulated in breast cancer tissues compared to non-cancerous tissues and a normal cell line, whereas miR-646 expression is decreased in breast cancer cells. The downregulation of HDAC2 and overexpression of miR-646 suppressed breast cancer cell proliferation [87].

Low levels of miR-125a were associated with the poor prognosis of patients with neuroblastoma [88]. miR-125a inhibited neuroblastoma (NB) cell proliferation by inducing apoptosis [88]. HDAC2 was highly expressed in NB tissues and cells and decreased miR-125a expression [88]. The inhibition of HDAC decreased CCAAT/enhancer-binding protein α mRNA expression in HCC cells by inducing miR-124–3p and miR-25 expression [89]. HDAC2 was upregulated, and miR-503–5p was down-regulated in ESCC [90]. HDAC2 bound to the promoter sequences of miR-503–5p. and miR-503–5p suppressed the proliferation of ESCC cells both in vitro and in vivo [90].

Breast cancer gene 1 (BRCA1) bound to HDAC2 and repressed oncogenic miR-155 expression [91]. The downregulation of miR-155 inhibited the growth of tumor cell lines in vivo [91]. Thus, miR-155 could serve as a target for developing drugs for BRAC1-deficinet breast cancers. miR-183 overexpression induced apoptosis and inhibited cell growth both in vitro and in vivo [92]. The neuroblastoma MYC oncogene MYCN recruited HDAC2 to miR-183 promoter sequences to decrease the expression of miR-183 in neuroblastoma cells [92]. The downregulation of proline, glutamate and leucine-rich protein 1 (PELP1) decreased the tumorigenic and metastatic potential of breast cancer cells [93]. The overexpression of miR-200a and miR-141 mimetics decreased the tumorigenic and metastatic potential of breast cancer cells [93]. PELP1 bound to miR-200a and miR-141 promoter sequences and regulated the expression of these miRNAs by recruiting HDAC2 [93]. Thus, PELP1 could regulate tumor metastasis by controlling the expression and functions of the tumor metastasis suppressors miR-200a and miR-141. These reports indicate that molecular network involving HDAC2, and miRNAs can serve as targets for developing anti-cancer drugs. Table 2 shows a molecular network involving HDAC2 and miRNAs.

**Table 2:** Molecular networks involving miRNAs and HDAC2.

miRNAs	Expression in Cancers	Functions/Mechanisms	Ref.
miR-31	↓ Hepatocellular carcinoma ↓ Gastric cancer	•Targets HDAC2 •Apoptosis	[78], [79]
miR-122-5p	↓ Hepatic stellate cells	•c-Abl/HDAC2 pathway	[80]
miR-145	↓ Hepatocellular carcinoma	•Targets HDAC2 •Suppression of tumor growth	[81]
miR-155	↓ Breast cancer	•Targets HDAC2 •↓ErbB2 signaling	[82]
miR-455-3p	↓ Hepatic cancer	•Targets HDAC2 •↑P53 signaling	[83], [84]
miR-490-3p	↓ Prostate cancer	•Targets HDAC2 •Suppression of cell proliferation	[85]
miR-500a-5p	↓ Colorectal cancer	•Targets HDAC2 •Suppresses p300/YY1/HDAC2 axis	[86]
miR-646	↓ Colorectal cancer	•Targets HDAC2 •Suppression of cell proliferation	[87]
miR-125	↓ Neuroblastoma	•Targeted by HDAC2 •Induces apoptosis	[88]
miR-503-5p	↓ Esophageal squamous cell carcinoma	•Targeted by HDAC2 •Suppression of cell proliferation	[90]
miR-155	↓ Hepatic cancer	•Targeted by BRCA1-HDAC2 complex	[91]
miR-183	↓ Neuroblastoma	•Targeted by MYCN-HDAC2 complex	[92]
miR-200a/-141	↓ Breast cancer	•Targeted by PELP1-HDAC2 complex	[93]

↓denotes decreased expression or activity. ↑denotes increased expression or activity. T bar arrows denotes negative regulation.

## 7 The role of HDAC2 in anti-cancer drug resistance

VPA induced apoptosis in drug-resistant mouse melanoma cell lines, including B16F10C and B16F10R [94]. Hyperphorylated form of HDAC2 was increased in B16F10R compared to drug-sensitive B16F10C [94]. Thus, HDAC2 might play a key role in anti-cancer drug resistance. The overexpression of small molecular glycoprotein serglycin (SRGN) was found in multidrug-resistant breast cancer cells [95]. The overexpression of SRGN could predict a poor response to chemotherapy [95]. SRGN, in association with YES-associated protein (YAP), conferred chemo resistance both in vitro and in vivo. The YAP/runt-related TF1 (RUNX1) complex induced chemo resistance by increasing the expression of HDAC2 [95]. NF-κB protected colon cancer cells from genotoxic stress in an HDAC2-dependent manner [96]. The inhibition of HDAC2 enhanced the sensitivity of glioblastoma cells to temozolomide by decreasing the expression of multidrug resistance protein 1 (MRP1) [97]. The inhibition of HDAC1 and HDAC2 but not HDAC3, HDAC6, or HDAC8 enhanced the sensitivity of chronic lymphocytic leukemia (CLL) cells to TRAIL-induced apoptosis [98].

High expression levels of FKBP3 and HDAC2 have been found in CRC tissues [99]. High levels of HDAC2 conferred resistance to oxaliplatin in CRC cells [99]. The downregulation of FKBP3 enhanced the sensitivity of CRC cells to oxaliplatin by decreasing the expression of HDAC2 and increasing the expression of PTEN [99]. The downregulation of HDAC2 enhanced the sensitivity of multi-drug-resistant CC cells to 5-fluorouracil (5-FU) and oxaliplatin (Oxa) [100]. The combination of the HDAC inhibitor suberoylanilide hydroxamic acid and 5-FU or Oxaliplatin decreased HDAC2 expression and induced apoptosis in HT-29 cells [100].

Epigenetic silencing mechanisms play important roles in the chemo-resistance of human cancers. Metastasis-associated protein (MTA), a component of the nucleosome remodeling deacetylation complex, was increased in docetaxel-resistant PC cells [101]. MTA recruited HDAC2 to the promoter sequences of nuclear receptor 4A1 to confer resistance to docetaxel [101]. High levels of HDAC2 could predict the poor prognosis of patients with HCC [102]. An increased expression of ATP-binding cassette subfamily B member 1 (ABCB1) was responsible for resistance to doxorubicin [102]. Pyruvate dehydrogenase kinase 1 (PDK1) expression was upregulated by HDAC2 and EZH2 [103]. PDK1 served as a target of miR-148a in breast cancer cells [104]. DNMT1 recruited EZH2 and the HDAC2 complex to the promoter sequences of miR-148a and decreased miR-148a expression, which led to doxorubicin resistance in breast cancer cells [103]. The downregulation of HDAC2 enhanced acetylation in the promoter sequences of miR-22–5p, which led to the increased expression of miR-22–5p [104]. miR-22–5p then enhanced the radio sensitivity of HCC [104]. HDAC2 bound to the promoter sequences of miR-182 to decrease the expression of miR-182 in acute myelogenous leukemia (AML) [105]. HDAC inhibition increased the expression of miR-182, which decreased the level of RAD51 to enhance the sensitivity to double-strand damage-inducing agents [105].

Panobinostat induced apoptosis in both K562 and imatinib-resistant K562 (IR-K562) cells [106]. The genetic deletion of HDAC2 enhanced sensitivity to imatinib (Gleevec) in K-562 cells [106]. YTH domain-containing protein 1 (YTHDC1) regulates the splicing, export, and stability of mRNA. A low expression of YTHDC1 could predict the poor prognosis of patients with ccRCC [9]. YTHDC1 inhibited the progression of ccRCC by downregulating annexin A1 (ANXA1)/mitogen activated protein kinase (MAPK) pathways. The YTHDC1/ANXA1 axis modulated the sensitivity of tyrosine kinase inhibitors such as sunitinib [9]. HDAC2 inhibitors enhanced the sensitivity of ccRCCs to tyrosine kinase inhibitors through the YY1/HDAC2 complex [9]. CAY10683, an inhibitor of HDACs, in combination with imatinib. exerted synergistic effects on imatinib resistance by inhibiting HDAC2 and the PI3K/Akt signal transduction pathway in chronic myelogenous leukemia (CML) cells with imatinib resistance [107].

**Fig. 3:**
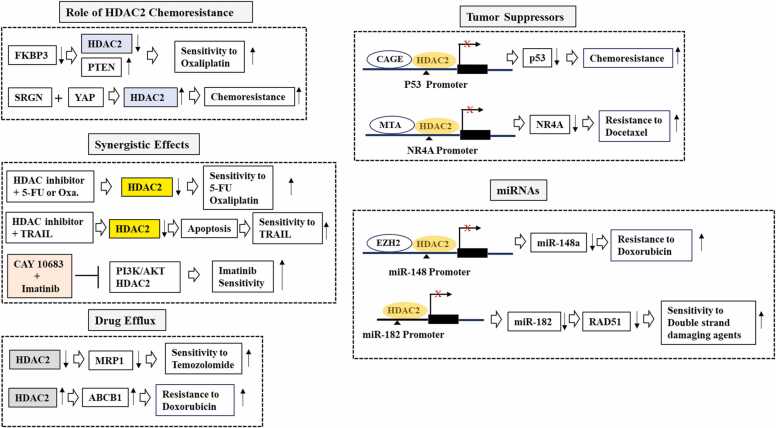
Mechanisms of HDAC2-promoted anti-cancer drug resistance. The hollow arrows denote direction of reaction. T bar arrows denotes negative regulation. The mechanisms of HDAC2-promoted anti-cancer drug resistance are as follows: 1) HDAC2 binds to CAGE, a cancer/testis antigen, to decrease the expression of p53, which results in resistance to various anti-cancer drugs; b) HDAC2 binds to the promoter sequences of miRNAs (miR-148a, miR-182) to decrease expression of these miRNAs, which results in anti-cancer drug resistance; 3) HDAC2 regulates expression of genes involved in drug efflux such as ABCB1 and MRP1 to confer anti-cancer drug resistance; 4) HDAC2 inhibition exerts synergistic effects to enhance sensitivity to TRAIL, imatinib, and chemotherapeutics. ↓denotes decreased expression or activity. ↑denotes increased expression or activity. X denotes suppression of transcription.

The expression of CAGE, a cancer/testis antigen, was increased in anti-cancer drug-resistant melanoma cells [108]. CAGE-HDAC2 complex bound to the promoter sequences of p53 to decrease the expression of p53 in anti-cancer drug-resistant melanoma cells [108]. The overexpression of CAGE conferred resistance to anti-cancer drugs in melanoma cells [108]. These reports indicate that HDAC2 could serve as a target to overcome resistance to various anti-cancer drugs. Fig. 3 shows the mechanisms of HDAC2-promoted anti-cancer drug resistance.

## 8 Conclusions

Many reports have indicated that HDAC2 could serve as a target for developing anti-cancer drugs. HDAC inhibitors have been developed as anti-cancer drugs. However, the development of anti-cancer drugs targeting HDAC2 has not been successful. Thus, it is necessary to develop anti-cancer drugs targeting HDAC2 by completely understanding the mechanisms of HDAC2-promoted cancer cell proliferation and anti-cancer drug resistance and the genes regulated by HDAC2. These genes can be employed as targets for developing anti-cancer drugs and will also make it easier to understand the mechanism of HDAC2-promoted cancer cell proliferation and anti-cancer drug resistance.

VPA was investigated in a clinical trial of 26 patients with progressing solid tumors [109]. Grade 3 or 4 neurological side effects occurred in 8 out of 26 patients [109]. However, no apparent hematological toxicity was observed. VPA induced the hyperacetylation of histone in most patients [109]. A phase I clinical trial (NCT00246103) of VPA was completed. This trial measured the maximum tolerable dose, efficacy, and pharmacokinetics of VPA in 82 patients with solid tumor malignancies. The recommended phase II dose of VPA was 60 mg/kg/d when given by one-hour intravenous infusion twice daily for 5 days every 3 weeks (NCT00246103). Synergistic activity between VPA and epirubicin was observed at 0.5 mM VPA (NCT00246103). Anti-cancer drugs usually display systemic toxicity and low bioavailability. Therefore, it is necessary to develop delivery vehicles that can overcome problems associated with small molecular anti-cancer drugs.

It is known that miRNAs can regulate the expression of HDAC2 [78], [79]. These miRNAs can be developed as anti-cancer drugs in the form of miR-mimics or miR-inhibitors. Unlike small interfering RNAs (siRNAs), miRNAs target multiple genes. This may lead to off-target effects. Most clinical trials involving miRNAs are phase I or phase II trials. Thus, it is necessary to improve the delivery, pharmacokinetics, and pharmacodynamics of miRNAs.

We and others have reported the presence of CAGE (DDX53), a cancer/testis antigen, in the exosomes of cancer cells [110], [111]. CAGE mediated cellular interactions (cancer cells, mast cells, and macrophages) within the tumor microenvironment to enhance the tumorigenic potential of CC cells [110]. Given the fact that HDAC2 binds to CAGE, it is necessary to examine the presence of HDAC2 in the exosomes of cancer cells. If so, HDAC2 might mediate cellular interactions within the tumor microenvironment. It would also be interesting to examine the presence of HDAC2-targeting miRNAs in exosomes.

We previously reported that the CAGE-derived CAGE-binding peptide enhanced the sensitivity of cancer cells to anti-cancer drugs [112], [113]. This peptide inhibited the binding of CAGE to GSK3β, enhancing the sensitivity of melanoma cells to anti-cancer drugs [113]. The domain of HDAC2 that confers resistance to anti-cancer drugs should be identified. This information could be used to design peptides to overcome resistance to anti-cancer drugs. Although HDAC2 can bind to CAGE, the CAGE-binding domains of HDAC2 have not been reported yet. HDAC2-derived peptides corresponding to these domains can also be developed as anti-cancer drugs for HDAC2/CAGE-expressing cancers.

The binding partners of HDAC2 were identified using the STRING database [114]. These binding partners included Sin3A-associated protein 30 (SAP30), metastasis-associated protein 1 (MTA1), methyl-CpG-binding domain protein 2 (MBD2), DNMT1, REST corepressor 1 (RCOR1), YY1, tumor protein53 (TP53), and metastasis-associated protein 2 (MTA2). The Sin3A-HDAC2 complex was reported to regulate the cellular reprogramming process [53]. MBD was essential for pluripotency in mouse embryonal stem cells [115]. The CoREST/HDAC2 complex bound to the promoter sequences of NANOG to regulate the integrity of primordial germ cells [116]. Thus, it is necessary to identify the HDAC2 domains that are critical for the binding of HDAC2 to these partners. The roles of these HDAC2-binding partners in tumorigenesis and anti-cancer drug resistance have not yet been extensively investigated.

Although HDAC2-targeting inhibitors have shown some promising anti-cancer effects, clinical trials have not led to the discovery of successful anti-cancer drugs. Screening inhibitors based on the full-length HDAC2 structure may lead to the discovery of authentic HDAC2 inhibitors with anti-cancer effects. Extensive studies on the HDAC2 interactome might produce HDAC2-selective chemical/peptides with anti-cancer activities without off-target effects.

## CRediT authorship contribution statement

**Hyein Jo:** Data curation, Formal analysis. **Kyeonghee Shim:** Data curation, Formal analysis. **Han-Ul Kim:** Data curation, Formal analysis, Writing – review & editing. **Hyun Suk Jung:** Data curation, Formal analysis, Funding acquisition. **Dooil Jeoung:** Data curation, Formal analysis, Funding acquisition, Writing – original draft. All authors participated in Writing – review & editing and agreed to the final manuscript.

## Declaration of Competing Interest

The authors declare that they have no known competing financial interests or personal relationships that could have appeared to influence the work reported in this paper.
